# Mesenchymal stem cell-loaded porous tantalum integrated with biomimetic 3D collagen-based scaffold to repair large osteochondral defects in goats

**DOI:** 10.1186/s13287-019-1176-2

**Published:** 2019-03-05

**Authors:** Xiaowei Wei, Baoyi Liu, Ge Liu, Fan Yang, Fang Cao, Xiaojie Dou, Weiting Yu, Benjie Wang, Guoshuang Zheng, Liangliang Cheng, Zhijie Ma, Yu Zhang, Jiahui Yang, Zihua Wang, Junlei Li, Daping Cui, Wei Wang, Hui Xie, Lu Li, Feng Zhang, William C. Lineaweaver, Dewei Zhao

**Affiliations:** 1 Laboratory of Orthopedics, Affiliated Zhongshan Hospital of Dalian University, Dalian, Liaoning China; 2 Department of Orthopedics, Affiliated Zhongshan Hospital of Dalian University, NO. 6 Jiefang Street, Zhongshan District, Dalian, 116001 Liaoning China; 3 Department of Biomedical Engineering, Faculty of Electronic Information and Electrical Engineering, Dalian University of Technology, Dalian, China; 4 JMS Burn and Reconstructive Center, Jackson, MS USA

**Keywords:** Osteochondral defect, Porous tantalum, Bone marrow mesenchymal stem cells, 3D collagen-based scaffold, Tissue engineering, Osteochondral regeneration

## Abstract

**Background:**

The body is unable to repair and regenerate large area bone defects. Moreover, the repair capacity of articular cartilage is very limited. There has long been a lack of effective treatments for osteochondral lesions. The engineered tissue with biphase synthetic for osteochondral repair has become one of the hot research fields over the past few years. In this study, an integrated biomanufacturing platform was constructed with bone marrow mesenchymal stem cells (BMSCs)/porous tantalum (pTa) associated with chondrocytes/collagen membranes (CM) to repair large osteochondral defects in load-bearing areas of goats.

**Methods:**

Twenty-four goats with a large osteochondral defect in the femoral heads of the left hind legs were randomly divided into three groups: eight were treated with chondrocytes/CM-BMSCs/pTa, eight were treated with pure CM-pTa composite, and the other eight goats were untreated. The repair effect was assessed by X-ray, gross observation, and histomorphology for 16 weeks after the operation. In addition, the biocompatibility of chondrocytes/CM-BMSCs/pTa was observed by flow cytometry, CCK8, immunocytochemistry, and Q-PCR. The characteristics of the chondrocytes/CM-BMSCs/pTa were evaluated using both scanning electron microscopy and mechanical testing machine.

**Results:**

The integrated repair material consists of pTa, injectable fibrin sealant, and CM promoted adhesion and growth of BMSCs and chondrocytes. pTa played an important role in promoting the differentiation of BMSCs into osteoblasts. Three-dimensional CM maintained the phenotype of chondrocytes successfully and expressed chondrogenic genes highly. The in vivo study showed that after 16 weeks from implantation, osteochondral defects in almost half of the femoral heads had been successfully repaired by BMSC-loaded pTa associated with biomimetic 3D collagen-based scaffold.

**Conclusions:**

The chondrocytes/CM-BMSCs/pTa demonstrated significant therapeutic efficacy in goat models of large osteochondral defect. This provides a novel therapeutic strategy for large osteochondral lesions in load-bearing areas caused by severe injury, necrosis, infection, degeneration, and tumor resection with a high profile of safety, effectiveness, and simplicity.

## Background

Osteochondral lesions are common in orthopedics. Owing to a lack of effective treatments, these lesions easily lead to functional disability and have severe effects on patient quality of life [1]. Clinical repair of osteochondral defects has been studied since the late 1970s [2]. They are usually caused by trauma, necrosis, inflammation, degeneration, and tumor. Those repairing methods include debridement, bone marrow stimulation, osteochondral graft, and arthroplasty. Autologous osteochondral transplantation (OATS or mosaicplasty) is the main treatment for small area lesions. Its main limitation alongside with limited donor graft material and the donor site pathology is of being technically challenging and often resulting in uneven articular surface [3]. Arthroplasty is commonly used to substitute the severely diseased joints, such as congenital hip dysplasia and middle-late osteonecrosis of the femoral head. However, severe complications such as severe infection, and loosening and sinking of the prosthesis may lead to failure. The ideal bone regeneration materials most have good mechanical properties and induce osteoconductivity and osteoinduction. When the length of a bone defect is more than a critical size bone defect, spontaneous healing of the body is impossible during the lifetime unless some osteogenic, osteoconductive, or osteoinductive material is placed in or onto the defect [4]. At present, autologous bone grafting carrying blood vessels is one of the most effective methods of treating large bone defects, but it may cause complications and deformity of the donor site. To date, there has not been a satisfactory treatment for osteochondral defects. The integrated repair of large osteochondral defects, and even the whole articular surface, has become a hot research area.

One-phase scaffolds have been applied in the early studies for the repair of bone and cartilage defects combined with osteoblasts or chondrocytes. In the study of Alhadlaq et al., chondrocytes and osteoblasts induced from bone marrow mesenchymal stem cells (BMSCs) were packaged with a polyethylene glycol (PEG) hydrogel suspension and implanted into nude mice. The formation of cartilaginous and bone-like tissue was observed, but the biomechanical strength of the construction was still very low to support the effective regeneration of osteochondral tissue [5]. The engineered tissue with biphase synthetic for osteochondral repair has become one of the hot research fields [6]. Various advanced scaffolds have been preclinically applied in osteochondral defects in animal models [7]. In general, the biodegradable upper cartilaginous layer of the scaffold is of lower strength and the underlying subchondral layer of higher strength [8]. Although potentially the concept of 3D printing could overcome the difficulties of transitions from bone and cartilage and construct integrated scaffolds, current 3D printing is restricted by the types of materials that can handle. Relevant research is mostly focused on the use of hydroxyapatite, PGA, PLA, PLGA, PCL, and other polymer materials. For example, a biphasic scaffold consisting of a PGA/PLA scaffold and a PCL/HA scaffold has been reported in the regeneration of goat femoral heads [9]. Chondrocytes or BMSCs were seeded into the scaffolds for cartilage and bone reconstruction, respectively. Ten weeks after subcutaneous implantation in nude mice, the cell-scaffold constructs successfully regenerated goat femoral heads, but the reconstituted femoral head was not implanted in goats. In addition, the decomposition products of PGA/PLA were acidic, so the long-term use of these materials remains to be validated. In contrast, degradable materials consisting of natural cartilage components, such as collagen or proteoglycan, have obvious advantages in long-term clinical applications. In reality, the extracellular matrix of cartilage is a structurally complex 3D environment that even highly sophisticated and newly developed biomaterials will probably never reach this complexity [10]. However, it is noteworthy that the natural polymers possess more favorable biocompatibility, but less controllable compared to the synthetic polymers.

Tantalum metal has good biocompatibility, ductility, and tenacity. It is also highly resistant to corrosion and does not result in body tissue stimulation. Human mesenchymal stem cells cultured on tantalum plates exhibit good osteogenic differentiation [11]. In addition, solid tantalum (sTa) is beneficial for the attachment, growth, and differentiation of human osteoblasts [12]. It is reported that porous tantalum (pTa) with a reticulated vitreous carbon (RVC) scaffold has a stable and ideal connection and integration with host bone in dogs [13]. PTa, in combination with periosteal grafts, promotes excellent bone incorporation of the scaffold into trabecular subchondral bone and vice versa [14] (Table 1).

**Table 1:** Mechanical properties of porous tantalum (pTa), solid tantalum (sTa), and bone tissues

Sample	All pore mean size (μm)	Open porosity (%)	Tantalum coating thickness (μm)	Compressive strength (MPa)	Young’s modulus (GPa)
pTa	200–500	75–85	15–30	30–90	10–30
sTa				Not available	186–191
Cortical bone				100–230	7–30
Cancellous bone				2–12	0.01–3

*pTa* porous tantalum, *sTa* solid tantalum

Chondrocytes around the bone defect area are surrounded by the cartilage matrix. Hence, the ability of proliferation and migration is limited. Moreover, healing of the cartilage is difficult to achieve; one of the most important reasons is its avascular structure. Cartilage lesions (9 mm or greater) have been reported to be biomechanically unstable with a high propensity of progression to degenerative joint disease [15]. The use of debridement, grinding, drilling, and cartilage cell therapy may form fibrous cartilage because of mechanical defects, thus ultimately leading to degenerative disease of the joints. Transplantation with allografts or autografts can also be used to repair chondral and osteochondral defects. However, several disadvantages of allograft transplantation include disease transmission, immune reaction, and slower remodeling [16]. In addition, autograft transplantation is also disadvantageous because it requires patients to undergo multiple surgeries, and it is also associated with donor site morbidity. For the repair of articular cartilage, the biodegradable materials from tissue engineering are currently and commonly used [17]. Cartilage primarily consists of water, collagen, proteoglycans, chondrocytes, and a few minor proteins. Therefore, it has become interesting to study the natural ingredients, such as collagen and proteoglycan. In the study by Xia et al., pericellular collagen I coating facilitated the homing of MSCs to the full-thickness cartilage lesion in a rabbit model and their differentiation toward the chondrocytes, which is beneficial for the regeneration of cartilaginous tissue [18]. Munir et al. used PCL/collagen scaffolds for cartilage tissue engineering, they found that 0.2% collagen I presented to have compressive properties similar to that of the native cartilage at 10% strain [19].

In this study, we have successfully fabricated pTa, which was prepared through the chemical vapor deposition (CVD) technique on the porous silicon carbide (SiC) scaffold for the first time. We established a large osteochondral defect model in the load-bearing areas of femoral heads. Integrated repair for almost half of the femoral heads defect was finished successfully by BMSC-loaded pTa combined with biomimetic 3D collagen-based scaffold in large animals with a safe, effective, and simple method.

## Materials and methods

### Isolation, culture, and identification of BMSCs

BMSCs were isolated from the marrow of the femoral condyles of 10-month-old goats. The bone marrow was centrifuged with heparin, pooled and resuspended in DMEM/F12 supplemented with 10% FBS, 100 UI/mL penicillin, and 100 μg/mL streptomycin. After 2 days, cultures were carefully rinsed with phosphate buffer saline (PBS) to remove non-adhered cells and cultured in a fresh culture medium.

For flow cytometry, 5 × 10^5^ cells in 100-μL PBS were incubated with 5-μL PE-conjugated rat anti-goat CD44, 5-μL FITC-conjugated mouse anti-goat CD14, 5-μL Alexa Fluor 405-conjugated mouse anti-goat CD79a, or 5-μL Alexa Fluor 405-conjugated mouse anti-goat HLA-II for 15 min, respectively. The negative control was generated by replacing the antibody with different isotype IgG. For CD29, we use 5-μL mouse anti-goat CD29 and 5-μL FITC-conjugated rabbit anti-mouse secondary antibody. After washing with PBS containing 1% BSA, the fluorescence of cells was analyzed using a Coulter EPICS XL flow cytometer.

The multiple lineage differentiation of BMSCs was evaluated by a tri-lineage differentiation experiment toward osteogenesis, adipogenesis, and chondrogenesis. Briefly, BMSCs at passage 4 were used for tri-lineage-induced experiments. Goat BMSCs in passage 3 were harvested and seeded in 6-well plates at a density of 5 × 10^4^ cells/well. After confluence in growth medium, the cells were treated with *osteogenic induction medium* for BMSCs identification [20]. Alkaline phosphatase and alizarin red staining were performed at 14 and 21 days during the induction culture. When the ALP staining was performed, the medium was removed, and the cell layers were rinsed with PBS 3 times and fixed in 4% paraformaldehyde for 20 min at RT. Then, the fixed cells were incubated with an NBT/BCIP ALP Color Development Kit for 30 min at 37 °C. For alizarin red staining, the fixed cells were incubated with 0.1% alizarin red S for 30 min at 37 °C. For adipogenesis, the goat BMSCs (5 × 10^4^ cells/well) were cultured in a 6-well plate with BMSC adipogenic differentiation medium [21]. The cells were evaluated for adipogenesis by oil red O staining after 21 days in culture. For chondrogenesis, a standard pellet culture was performed. For chondrogenic differentiation, the cell sheets were manually agitated at the edges and aggregated into pellets following a protocol [22]. After 3 weeks of chondrogenic incubation, the pellet was formalin-fixed and paraffin-embedded. Alison blue staining was then performed to assess the chondrogenic capacity of the BMSCs.

### Isolation, culture, and identification of chondrocytes

The chondrocytes were isolated from articular cartilage from the knees of 10-month-old goats. Chondrocytes were isolated, cultured, and expanded DMEM supplemented with 10% FBS, 100 UI/mL penicillin, and 100 μg/mL streptomycin, 4 mM glutamine, 0.1 mM nonessential amino acids, 0.4 mM proline, 1 mM sodium pyruvate, and 50 μg/mL ascorbic acid according to previously reported methods [23]. The cell culture medium was refreshed every 3 days.

Chondrocytes in passage 2 were harvested and fixed in 4% paraformaldehyde for 1 h at RT. Then, the fixed cells were incubated with 1% toluidine blue for 2 h at 37 °C. Cells cultured in normal growth medium served as the control.

Meanwhile, the chondrocytes were identified by immunocytochemistry. Cells were fixed and incubated with 0.1% Triton X-100 for 20 min. Slides were incubated in 3% H_2_O_2_ in methanol for 10 min to block endogenous peroxidase activity. After washing in PBS, slides were blocked with blocking buffer supplemented with normal serum at RT for 10 min to eliminate non-specific binding of conjugated secondary antibodies before incubation overnight at 4 °C with a collagen II antibody. After several rinses with PBS, slides were incubated with a biotinylated goat anti-mouse immunoglobulin. The signal was visualized with peroxidase-labeled streptavidin-complexed DAB, and the sections were briefly counterstained with hematoxylin. The negative control was generated by replacing the primary antibody with PBS.

### Production of three-dimensional pTa scaffold and sTa using CVD

A pTa scaffold was prepared as our previously established method [24]. The porous SiC substrate was supplied by the Institute of Metal Research, Chinese Academy of Sciences, which was prepared by polymer pyrolysis combined with a liquid infiltration-reaction process. Tantalum deposition on porous SiC and solid SiC was achieved using a low-temperature CVD system.

### Cytotoxicity of the pTa by CCK8

The extract was prepared as follows. According to international standard ISO 10993-12 [25], the pTa sample (Ta-SiC) and porous SiC sample were sterilized. BMSCs in passage 3, chondrocytes, and osteoblast cell line MG63 from ATCC were cultured and used to assess the cell toxicity of the porous Ta-SiC and porous SiC. 5 × 10^4^ cells/mL were seeded in the wells of a 96-well plate. Cells were cultured at 37 °C for 1 h, and then, an autoclaved pTa or porous SiC was added with sterile forceps to each well. In the control group, there was no any porous scaffold, only 5 × 10^4^ cells/mL were seeded in the wells of a 96-well plate. Then, 100-μL fresh culture medium with a 10-μL CCK8 solution was added to each well. After a 2-h incubation, the absorbance of each well was measured at 450 nm on a Bio-Rad America microplate reader.

### Characteristics of the CM-pTa scaffold and cell adhesion in vitro

Biodegradable three-dimensional collagen membranes (3D CM) clinically used for cartilage repair were purchased from Geistlich Pharma AG, Switzerland. The amicrobic 3D CM and pTa were submerged in PBS to reduce liquid rejection by electrostatic loading. 1 × 10^6^ cells/mL BMSCs (or chondrocytes) in suspension culture medium was loaded onto pTa (or 3D CM). After 7 days of co-culture, pTa and 3D CM with different kinds of cells were rinsed with PBS and fixed with 2% glutaraldehyde for 2 h. Then, the two materials were infiltrated in dehydrated alcohol with a concentration gradient. Samples were treated with a critical point dryer, and one surface of each sample was spattered with gold in vacuum. The microstructure and composition of the scaffold were evaluated by SEM in a JEOL (Tokyo, Japan) JSM-6360LV instrument to yield an energy spectrum.

### Mechanical testing

Mechanical testing of cylindrical porous samples was carried out in accordance with the standard ISO 13314 [26]. All tests were carried out using a Hung Ta HT-2402 mechanical testing machine (Taiwan, China). Three rectangular 3D CM were in tension by applying a constant deformation rate of 1 mm/min. Mechanical testing was performed on parts that were 8 mm in width and 0.5 thickness; three round 3D CM in compression by applying a constant deformation rate of 0.5 mm/min. Mechanical testing was performed on parts that were 15 mm in diameter; three cylinders of pTa were tested in compression by applying a constant deformation rate of 0.5 mm/min. Mechanical testing was performed on parts that were 8 mm in diameter and 10 mm high; the combining power of the interface between 3D CM and pTa with injectable porcine fibrin sealant was represented as tensile strength. Three cylindrical samples were tested in the tension of interface by applying a constant deformation rate of 1 mm/min. Both sides of each CM were combined with one pTa cylinder by injectable porcine fibrin sealant. Each pTa cylinder was 10 mm in diameter and 10 mm high; The shear stress of the interface between 3D CM and pTa with injectable porcine fibrin sealant was represented as compressive strength. Three cuboid samples were tested in compression of the interface by applying a constant deformation rate of 0.5 mm/min. Both sides of each pTa cuboid were combined with one CM by injectable porcine fibrin sealant. Then, these two CMs were combined with two new pTa cuboids on the other side by injectable porcine fibrin sealant. Each cross section of pTa cuboids was 18 mm in length and 10 mm width.

### Total RNA extraction, cDNA synthesis, and Q-PCR analysis

3 × 10^5^ cells/mL BMSCs in suspension culture medium were loaded onto sTa or pTa surfaces in basic medium for 7, 14, and 21 days. For mRNA expression of osteogenic genes, the sTa group was considered as control. To evaluate the mRNA expression, adherent cells on each specimen were lysed with RNAiso Plus (Takara, Tokyo, Japan) according to the manufacturer’s instructions.

For mRNA expression of chondrogenic genes, 2D CM group was considered as control. In order to detect the change of mRNA in chondrocytes which culture with biodegradable, two-dimensional collagen membranes (2D CM) or 3D CM, 8 × 105 cells/mL chondrocytes in suspension culture medium were loaded onto 2D CM or 3D CM for 5 days. 3D CM for cartilage repair were purchased from Geistlich Pharma AG, Switzerland. 2D CM were prepared with collagen powder (Sigma-Aldrich, St. Louis, MO, USA).

Contaminating DNA in RNA preparations was removed with DNase I. Three independent experiments were performed, using separate samples of BMSCs (or chondrocytes) for RNA isolation. The RNA was reversely transcribed with reverse transcriptase according to the manufacturer’s instructions (Takara, Tokyo, Japan). The cDNA preparations were then cleaned by ethanol precipitation, and the cDNA pellets were diluted in an appropriate amount of RNase-free H_2_O. Q-PCR was performed on a 7500 Fast Q-PCR system (Applied Biosystems) for alkaline phosphatase (ALP), Osterix (OSX), osteocalcin (OCN), collagen I (Col-I), osteonectin (OSN), RUNX2, collagen II (Col-II), aggrecan (Agg), SOX9, collagen-X (Col-X), and GAPDH. Selection of primers for ALP, OSX, OCN, Col-I, OSN, RUNX2, Col-II, Agg, SOX9, Col-X, and GAPDH was performed using the Primer 5 (see Table 2). Q-PCR was performed in triplicate using SYBR® *Premix Ex Taq™* II Kit (Takara, Tokyo, Japan). The two-step PCR conditions were set up. The housekeeping gene GAPDH was used as the internal control gene to normalize the quantities of target genes. Relative mRNA abundance was determined by the 2^−ΔΔCt^ method and reported as fold induction.

**Table 2:** Sequences of oligonucleotide primers used to amplify the listed mRNA species, and PCR product sizes

Target gene	Primer sequence	Annealing temperature (°C)	Product size (bp)
ALP	F:tacttgtgcggggtgaagg	60	144
	R:cacgatgcccacggattt		
OSX	F:ggcaaagcaggcacaaaga	60	184
	R:ctgggaacgagtgggaaaag		
OCN	F:cagcgaggtggtgaagaga	60	144
	R:tggaagccgatgtggtcag		
Col-I	F:tggtgaagcaggcaaacct	60	87
	R:aaacctctctcgcctcttgct		
OSN	F:tcgactcttcctgccacttct	60	200
	R:gttgtcctcgtccctctcgt		
RUNX2	F:ccgaaatgcctctgctgtt	60	87
	R:aaactcttgcctcttccactcc		
Col-II	F:ctcaagtccctcaacaaccaga	60	123
	R:ccagtagtctccgctcttcca		
Agg	F:cctcaccatcccctgctactt	60	119
	R:gcaccacctccttctccttg		
SOX9	F:caagttccccgtctgcatc	60	111
	R:tgcggcttgttcttgctc		
Col-X	F:gaacggcacccctgtaatgt	60	99
	R:tggtcgttctcggtgaggt		
GAPDH	F:ttgtgatgggcgtgaacc	60	127
	R:ccctccacgatgccaaa		

### Repair of large osteochondral defects in the load-bearing areas of femoral heads

**Scheme 1:**
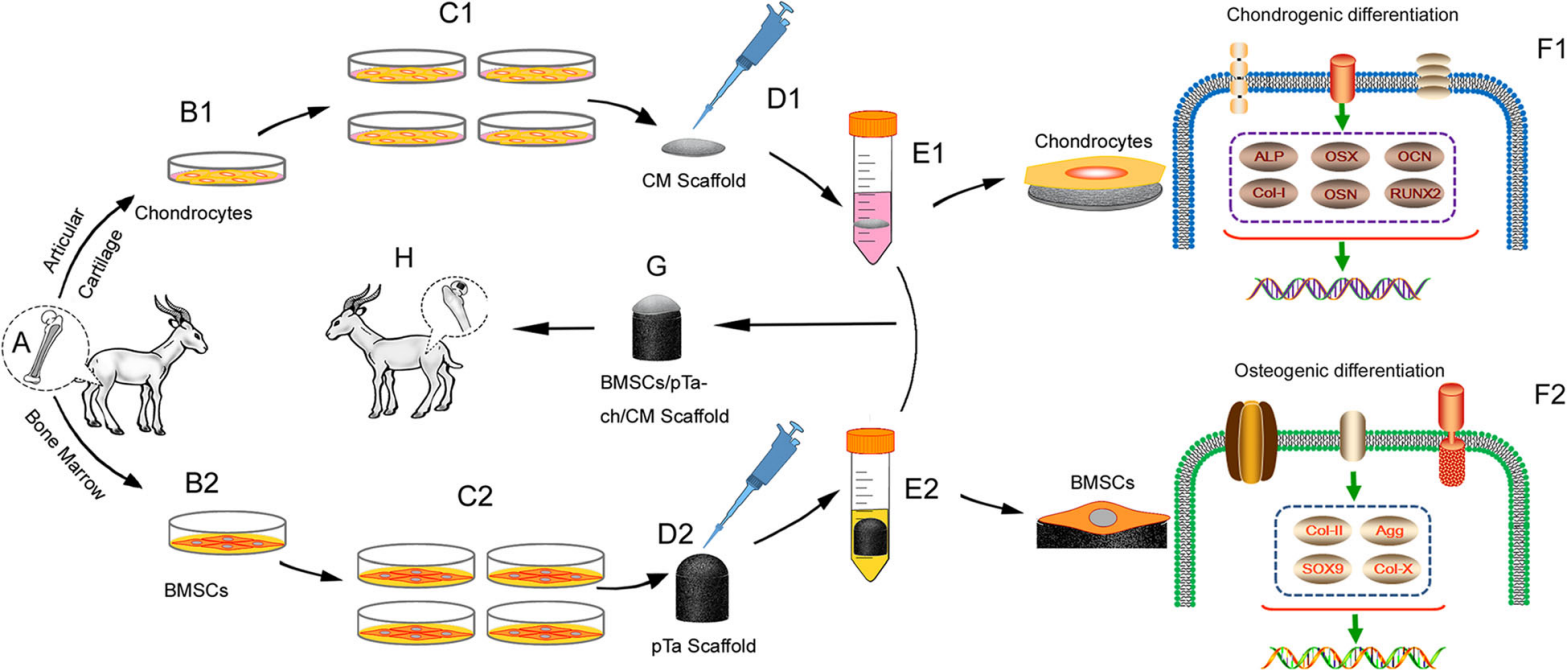
Schematic for the repairing of large osteochondral defects in goats’ femoral head with BMSCs/pTa-chondrocytes/CM biphasic scaffold. Chondrocytes and BMSCs were harvested from goat femora and femoral condyle, respectively (**A**). Chondrocytes (**B1**) and BMSCs (**B2**) were proliferated in vitro (**C1**, **C2**). Cartilaginous and osseous component scaffold were seeded with chondrocytes and BMSCs, respectively (**D1**, **D2**) and co-culture (**E1**, **E2**). mRNA expression of chondrogenic genes (**F1**) and osteogenic genes (**F2**). The cartilaginous and osseous component were combined with the fibrin sealant to form osteochondral construct (**G**), which was implanted into the femoral head of the homologous goat for 16 weeks (**H**)

This experimental protocol was performed according to the China Animal Research Guidelines, and all procedures on animals were approved by the Animal Ethics Committee of Dalian University. All animal subjects received humane care in compliance with the “Guide for Care of Laboratory Animals,” as detailed by the National Ministry of Science. Twenty-four 10-month-old goats were used in this study. Goats were randomly divided into three groups (*n* = 8). The left hind legs of the different experimental groups were used for the osteochondral defect model in femoral heads. The general experimental protocol is shown in Scheme 1. All surgeries were performed under anesthesia, and all efforts were made to minimize animals suffering. Goats were anesthetized with 3% pentobarbital (30 mg/kg), the femoral heads of the left hind legs were exposed. The cartilage defect was made by an untoothed ring drill. Sodium chloride (0.9%) was added to prevent necrosis of surrounding cartilage tissue from local overheating. Then, a subchondral bone defect along the direction of the femur was formed by a toothed ring drill. After flushing of the joint cavity, the osteochondral defect model (10.0-mm diameter and 12.0-mm depth) in femoral heads was established.

pTa cylinders (10-mm diameter and 10-mm height) with or without autologous BMSCs were implanted into subchondral bone defect sites. Sterile pTa rods and CM were placed in the wells of a 24-well plate. For a pTa rod containing BMSCs, 3 × 10^7^ cells/mL BMSCs were seeded onto the pTa rod. After 12 h, take away all the culture medium and rotate the pTa rod for 180°. Then 3 × 10^7^ cells/mL BMSCs were seeded onto the pTa rod again. 12 h later, the pTa rod with BMSCs was transferred to in a new well of 24-well plate. The cell culture medium was refreshed every 3 days and for 21 days in vitro before implantation. For the CM containing chondrocytes, 8 × 10^7^ cells/mL chondrocytes suspension was loaded onto the rough surface of CM. After 12 h, CM with chondrocytes was transferred to in a new well of 24-well plate. Chondrocytes were cultured with DMEM supplemented with 10% FBS, 100 UI/mL penicillin, and 100 μg/mL streptomycin, 4 mM glutamine, 0.1 mM nonessential amino acids, 0.4 mM proline, 1 mM sodium pyruvate, and 50 μg/mL ascorbic acid according to previously reported methods. The cell culture medium was refreshed every 3 days and for 21 days in vitro before implantation.

After pTa rods were implanted, an injectable porcine fibrin sealant (Guangzhou Bioseal Biotech, China) was placed on the tantalum rods. As soon as possible, the CM (10.0-mm diameter and 0.5-mm thickness) adhered to the tantalum rods with fibrin sealant. X-rays were used to determine the position of the tantalum at 16 weeks post-implantation. Sixteen weeks post-implantation, goats were anesthetized and sacrificed to gain femoral head samples. The ICRS score for gross observation was used to assess the integration of the scaffold into the joint [27].

### Histomorphology by hard tissue slicing and immunohistochemistry

Defect bearings were prepared for histological evaluation. Samples were fixed in 10% formaldehyde in phosphate buffer (pH 7.4). Femoral heads were dehydrated in a graded series of ethanol and embedded in plastic for hard tissue slicing. Ten-micrometer-thick sections were prepared throughout the defect/repair sites for Van Gieson’s stain. Five visual fields on each slice were selected randomly and imaged under × 100 magnification with an optical microscope. New bone formation areas in the pores of pTa were measured with ImageJ software. The specimens were evaluated by the Modified O’Driscoll histology score [27].

To analyze the chondrogenic potential of chondrocytes/CM scaffold in the femoral head, immunohistochemistry was performed using antibodies specific for collagen II (mouse antibody, dilution 1:500, Thermo Fisher Scientific, Waltham, MA, USA) and collagen I (rabbit antibody, dilution 1:500, Abcam, Cambridge, MA, UK) and Polink-2 plus® Polymer HRP Detection System Kit (ZSGB-BIO, Beijing, China). The negative control was generated by replacing the primary antibody with PBS. Slides were mounted and visualized at × 10 magnifications on an inverted microscope (Olympus, Tokyo, Japan). Image-Pro Plus 6.0 software was used to analyze quantitatively and to calculate the mean density. Five random regions in each section (three sections in each group) were selected, and the mean density was calculated as the ratio of IOD to the selected area.

### Statistics

Each experiment was repeated three times, and the results are presented as mean ± SD. For CCK8 test, six replicates were used for each sample to obtain a mean value. The significant differences between test groups were analyzed by one-way ANOVA and LSD post hoc test. *P* values < 0.05 were considered statistically significant. Differences in histological scoring between groups were analyzed using Wilcoxon rank-sum tests.

## Results

### Identification of BMSCs and chondrocytes

**Fig. 1:**
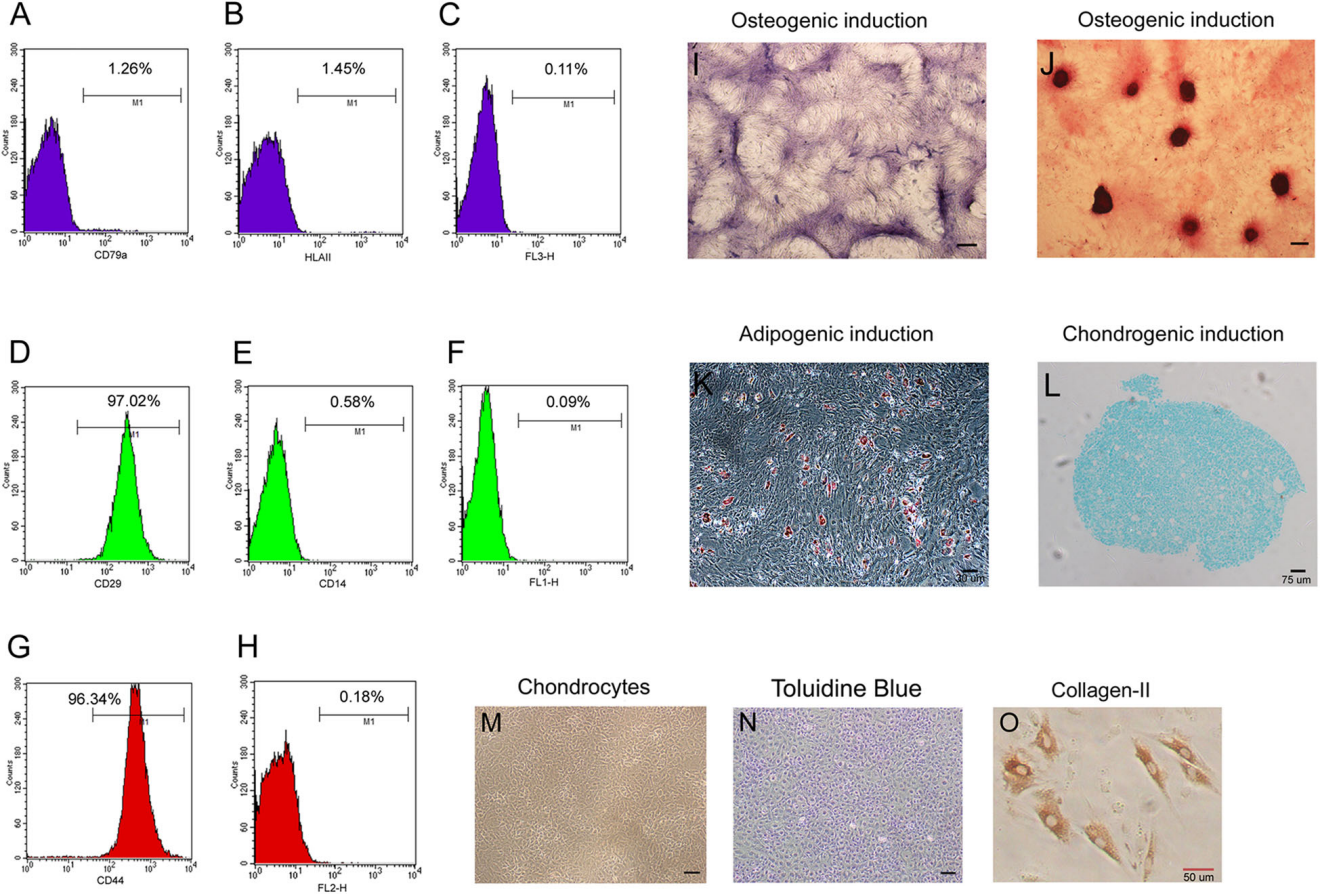
Identification of BMSCs and chondrocytes. BMSCs were identified with the surface markers **a** CD79a^−^ (1.26%), **b** HLA-II^−^ (1.45%), **d** CD29^+^ (97.02%), **e** CD14^−^ (0.58%), **g** CD44^+^ (96.34%). **c**, **f**, **h** The negative control was generated by replacing the antibody with isotype IgG. BMSCs in passage 3 cultured in osteogenic induction medium for (**i**) alkaline phosphatase were detected in the cytoplasm after induction of osteogenic differentiation for 14 days. **j** Alizarin red staining showed small calcium nodules in unlabeled after induction of osteogenic differentiation for 21 days. **k** Adipogenesis was examined using oil red O staining. **l** Alison blue staining was performed to assess the chondrogenic capacity. **m** Light microscopy of chondrocytes. **n** Toluidine blue staining of chondrocytes. **o** Immunocytochemistry of collagen II with chondrocytes. All black bar = 200μm

The isolated cells from the bone marrow expressed the BMSC markers CD29 and CD44 (Fig. 1d, g), but scarcely expressed CD79a, HLAII, or CD14 (Fig. 1a, b, e). Our results demonstrate that, in osteogenic medium, extracted cells from the bone marrow expressed alkaline phosphatase at 14 days (Fig. 1i). At 21 days, calcified nodules were detected by alizarin red staining in osteogenic medium, thus suggesting that the extracted cells entered the matrix mineralization period (Fig. 1j). Intracellular lipid droplets were observed after induction of adipogenesis for 21 days (Fig. 1k). Alison blue staining to assess the chondrogenic capacity was positive after induction for 21 days (Fig. 1l). All of these results confirmed that the primary cells extracted from the bone marrow primarily consisted of BMSCs. Chondrocytes in passage 2 demonstrated short fusiform or polygonal, closely arranged cells that were similar to irregular cobblestones (Fig. 1m). Strong positive toluidine blue staining conformed to the characteristics of chondrocytes (Fig. 1n). Moreover, most of the cells expressed collagen II which is considered as chondrocyte-specific expression protein by immunocytochemistry (Fig. 1o).

### Proliferation of BMSCs on pTa or SiC scaffolds

**Fig. 2:**
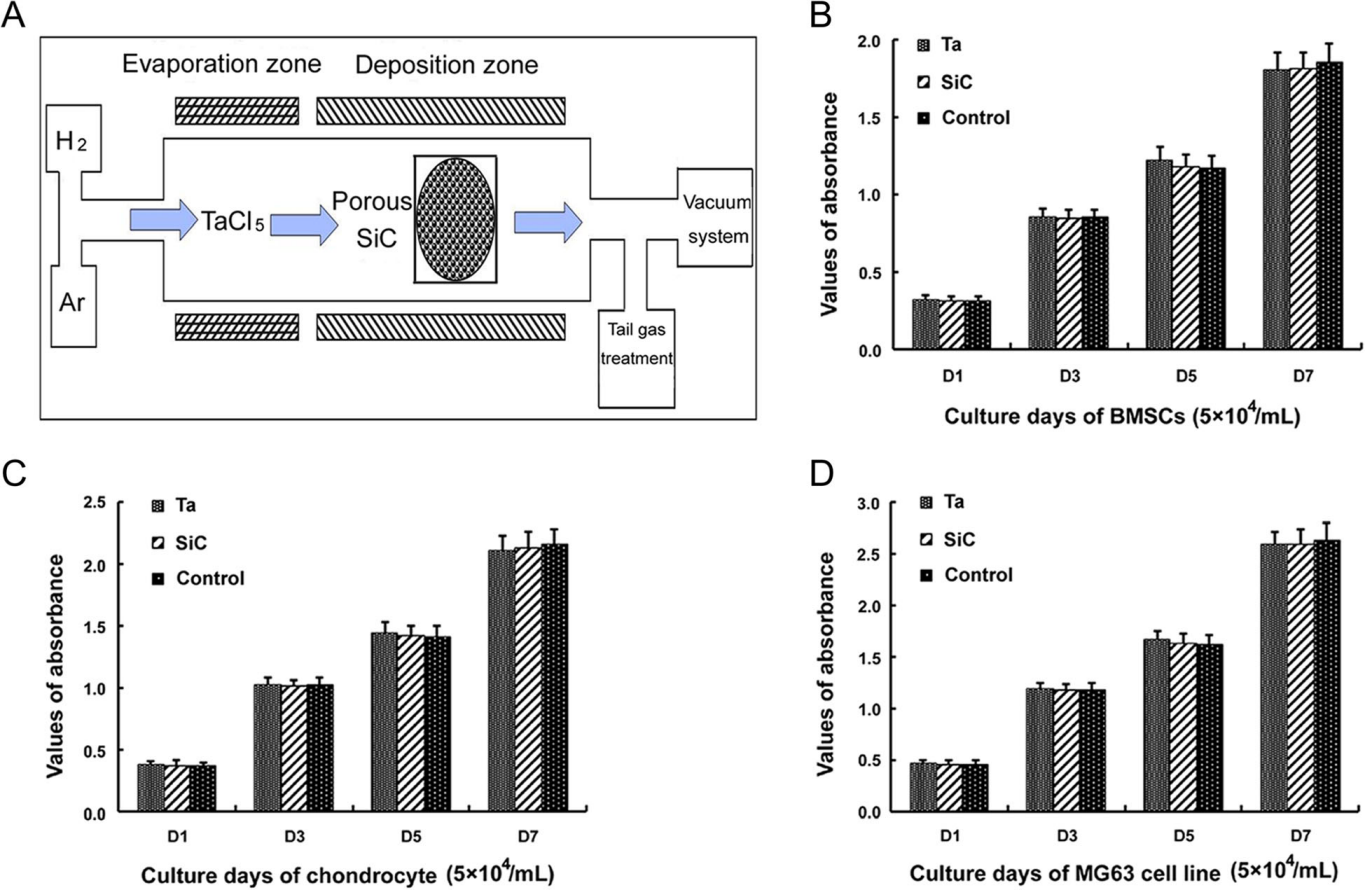
Process flow of pTa preparation and cytotoxicity test. **a** The schematic diagram of CVD methods. Proliferation of different cells co-culture with pTa or SiC scaffold. 5 × 10^4^/mL BMSCs (**b**), chondrocytes (**c**), or MG63 cells (**d**) were seeded in 96-well plates with DMEM/F12 medium, respectively. The proliferation of cells was assessed by CCK8 assay for 1, 3, 5, and 7 days (measures of optical density at 450 nm)

PTa scaffolds were prepared according to diagrammatic drawing (Fig. 2a). BMSCs in passage 3, chondrocytes, and MG63 cells were used for cytotoxicity assays of the pTa or SiC. The statistical results of CCK8 demonstrated that the proliferation of all types of cells co-cultured with pTa or SiC for 1, 3, 5, and 7 days was not inhibited, as compared with the control group (*P* > 0.05) (Fig. 2b, c, d). We determined that the pTa and SiC were not cytotoxic to BMSCs, chondrocytes or MG63 cells.

### Characteristics of the CM-pTa scaffold and cell adhesion in vitro

**Fig. 3:**
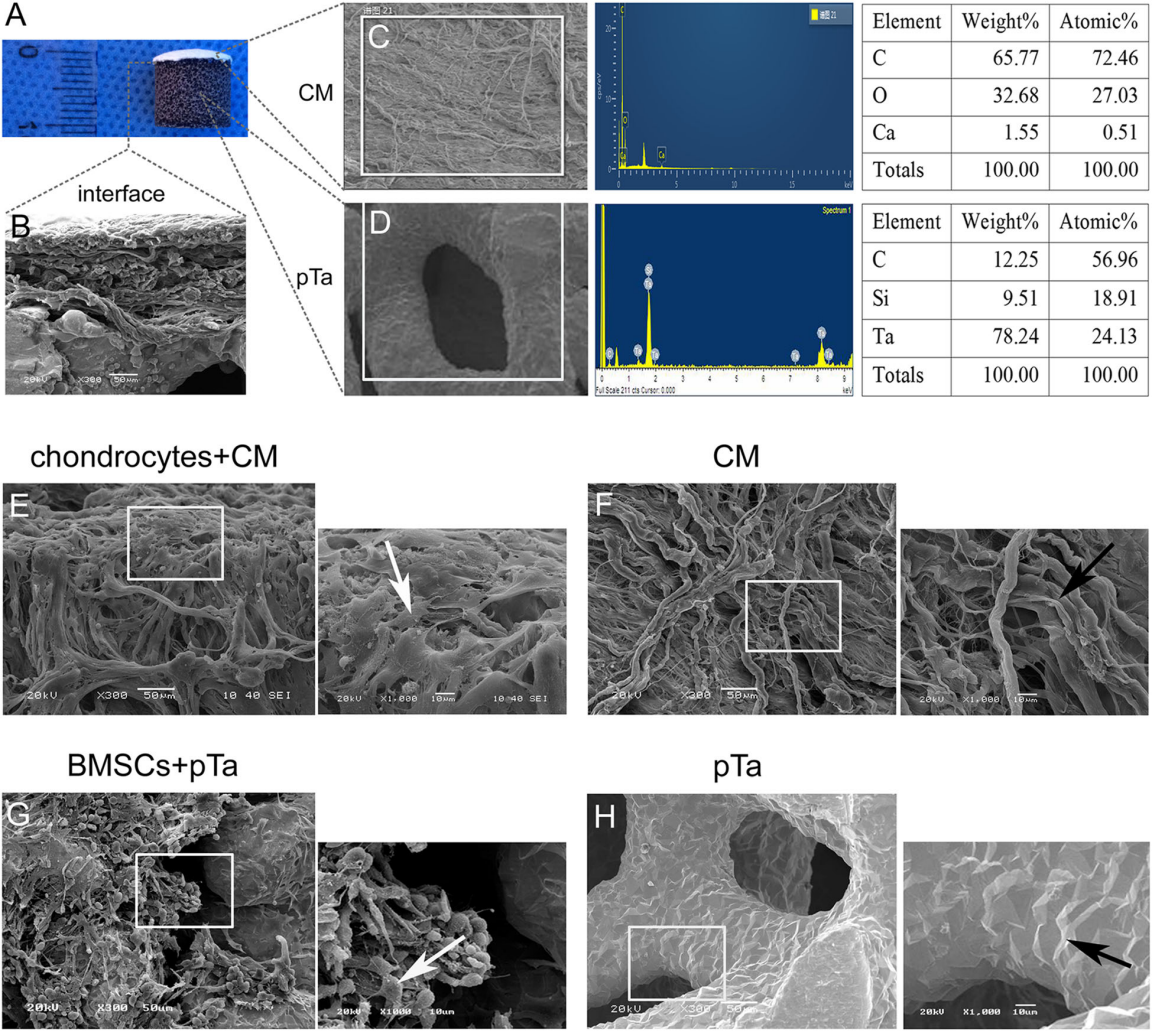
Characteristics of the CM-pTa scaffold and cell adhesion in vitro*.* **a** The appearance of BMSCs/pTa-chondrocytes/CM biphasic scaffold, **b** the interface between pTa and CM, **c** the component of CM, and **d** pTa analyzed by energy spectrum. **e** Morphology of chondrocytes cultured on CM, **f** CM control, **g** BMSCs cultured on pTa, and **h** pTa control was observed by SEM. For panels **e**, **f**, **g**, and **h**, big image was magnified × 300, bar = 50μm; small image was magnified × 1000, bar = 10μm

**Fig. 4:**
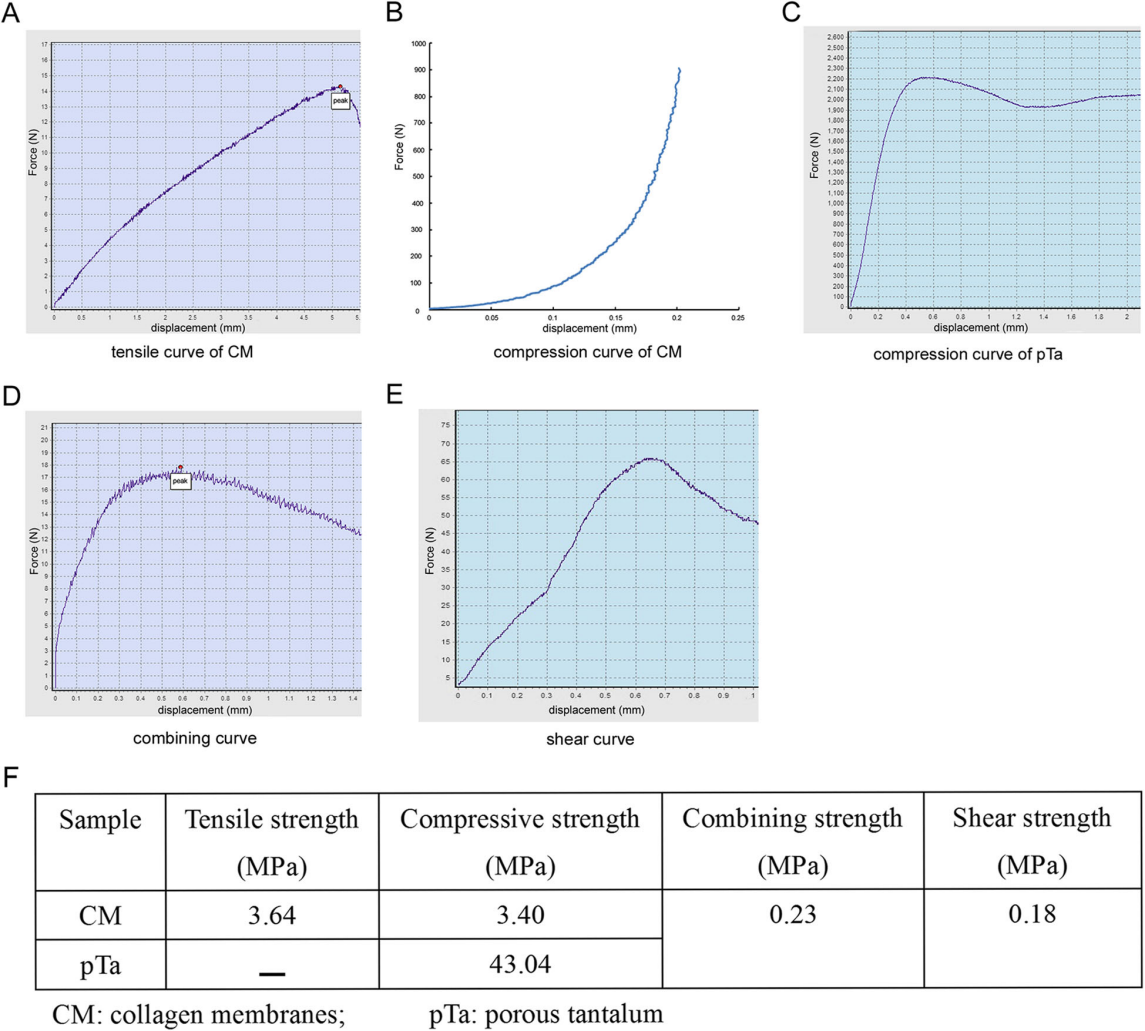
Mechanical properties of 3D CM, pTa scaffold, and their interface. **a** Tensile curve of 3D CM, **b** compression curve of 3D CM, **c** compression curve of pTa, **d** combining curve of the interface between 3D CM and pTa with injectable porcine fibrin sealant, **e** shear curve of the interface between 3D CM and pTa with injectable porcine fibrin sealant, and **f** mechanical properties of each layer

PTa and CM were fixed with injectable porcine fibrin (Fig. 3a). SEM results demonstrated that the interface was uniform and smooth, with firm adhesion (Fig. 3b). Energy spectrum analysis confirmed that CM was composed of C, O, and Ca elements only, and the weight percentages were 65.77%, 32.68%, and 1.55%, respectively (Fig. 3c). In addition, our pTa consisted of C, Si, and Ta, and the weight percentages were 12.25%, 9.51%, and 78.24%, respectively (Fig. 3d). It was very important for the cells to adhere to the material surface during the first step, and this process had large effects on the subsequent proliferation and differentiation of cells. The distribution pattern of cells in material pores may affect the tendency of osteogenic differentiation in vivo. On the 7th day of co-culture in vitro, the morphology and adhesion of chondrocytes or BMSCs were observed via SEM (Fig. 3e, g). The results demonstrated that chondrocytes were distributed evenly in the interior and the surface of CM. Adherent chondrocytes made contact with one another and completely covered the scaffold. Adherent chondrocytes were flat and fully stretched in polygonal shapes with long pseudopodia (Fig. 3e). The pores of the CM were filled with chondrocytes and the secreted matrix. In the control group, filamentous collagen was observed to be uniform, and rough surface structures were clearly visible at high magnification (Fig. 3f). On the 7th day of co-culture of BMSCs and pTa in vitro, the SEM results demonstrated that most of the pores were evenly filled with BMSCs (Fig. 3g). BMSCs were round, oval, or fusiform in the three-dimensional environment. A large amount of matrix secretion was visible around the BMSCs. In the control group, the three-dimensional structure of pTa was clear (Fig. 3h). The results of the mechanical tests are summarized in Fig. 4f. To simulate the stress in vivo, we tested the tension and compression separately. Average tensile strength and compressive strength of CM are 3.64 MPa and 3.40 MPa with three samples, respectively (Fig. 4a, b). Average compressive strength of pTa with three samples is 43.04 MPa (Fig. 4c). The combining power of the interface between 3D CM and pTa with injectable porcine fibrin sealant was represented as tensile strength for 0.23 MPa (Fig. 4d). The shear stress of the interface between 3D CM and pTa with injectable porcine fibrin sealant was represented as compressive strength for 0.18 MPa (Fig. 4e).

### Expression of osteogenic and chondrogenic genes

**Fig. 5:**
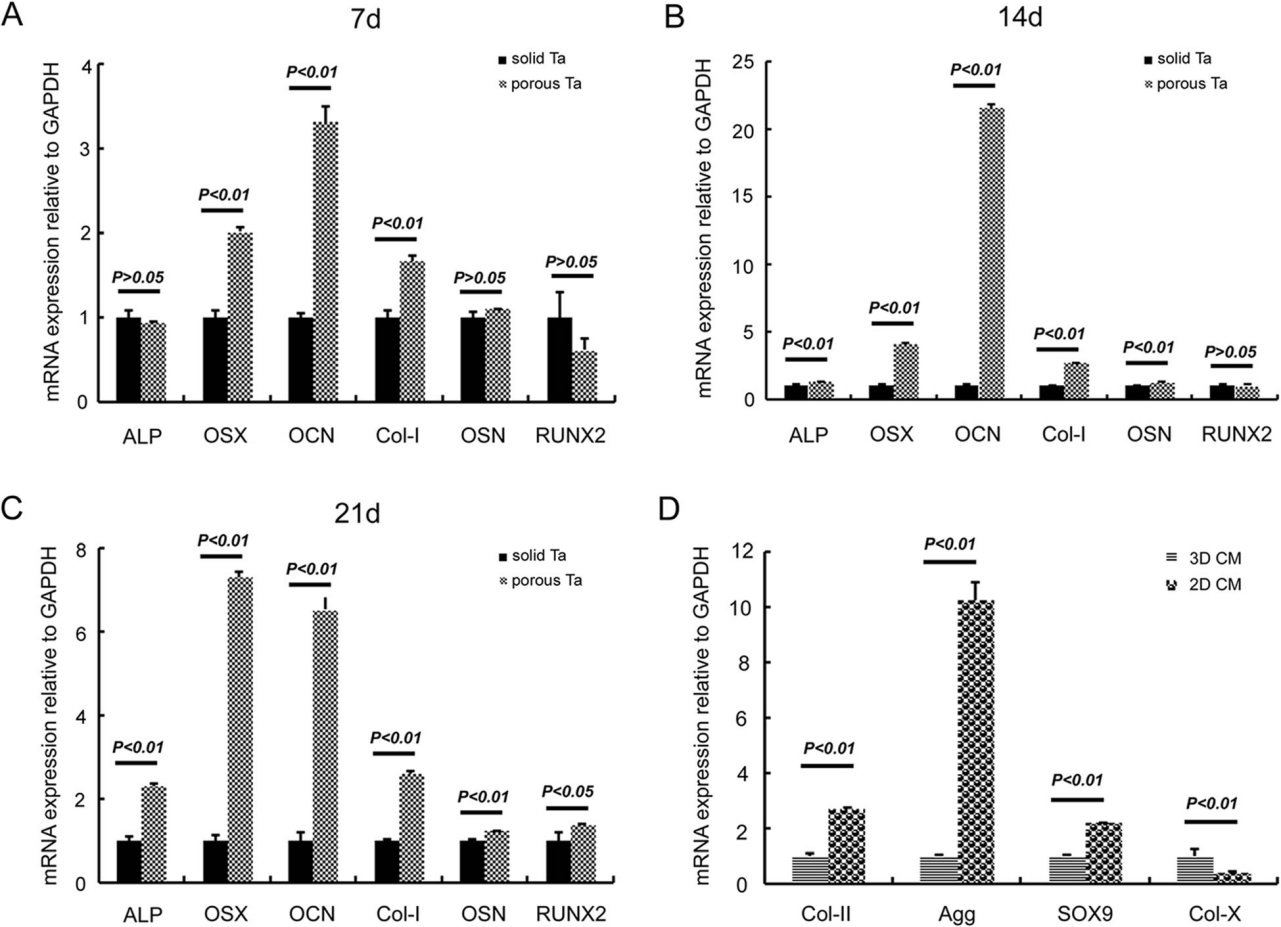
mRNA expression of osteogenic and chondrogenic genes. Comparison of mRNA transcript levels of bone-related genes in goat BMSCs cultured on solid tantalum (sTa) and porous tantalum (pTa) surfaces in basic medium for 7 days (**a**), 14 days (**b**), and 21 days (**c**). mRNA expression of cartilage-related genes in goat chondrocytes cultured on 2D CM and 3D CM in basic medium for 5 days (**d**)

Comparison of mRNA transcript levels of bone-related genes in goat BMSCs cultured on sTa and pTa surfaces. For a 7-day co-culture, significantly higher OSX, OCN, and Col-I levels were observed in pTa compared to sTa (*P* < 0.01) (Fig. 5a). For a 14-day co-culture, mRNA expression of ALP, OSX, OCN, Col-I, and OSN in pTa group was significantly higher than the expression in sTa group (*P* < 0.01) (Fig. 5b). After a 21-day co-culture, compared with sTa, 3D pTa can promote the expression of all the six osteogenic genes clearly, including RUNX2 (Fig. 5c). Moreover, mRNA expression of cartilage-related genes, such as Col-II, Agg, and SOX9 in goat chondrocytes cultured on 3D CM, was markedly higher than 2D CM group (*P* < 0.01). However, 3D CM can inhibit the expression of Col-X which is related to cartilage hypertrophy (*P* < 0.01) (Fig. 5d).

### Surgical procedures, imaging, and gross observation

**Fig. 6:**
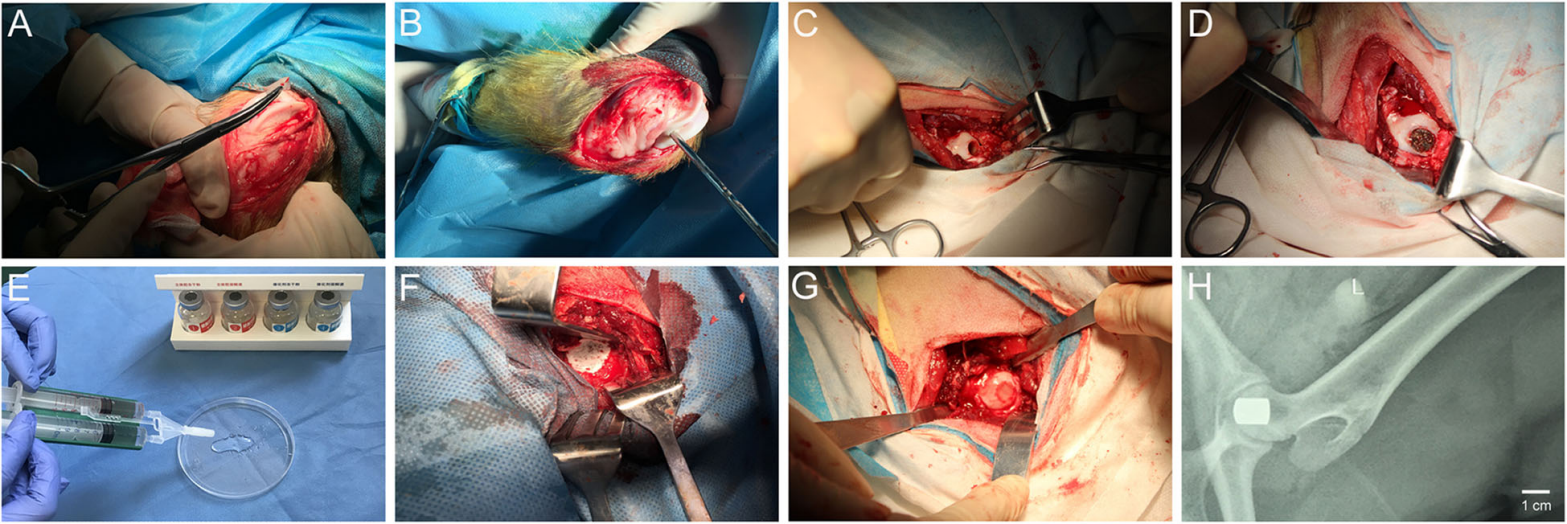
Stages of osteochondral defect creation and the surgical procedure of repairing osteochondral defect with BMSCs/pTa-chondrocytes/CM. **a** The chondrocytes were isolated from articular cartilage from the knees of 10-month-old goats. **b** BMSCs were isolated from the marrow of femoral condyle of 10-month-old goats. **c** Osteochondral defect model with 10.0-mm diameter and 12.0-mm depth in femoral heads was established. **d** The 10.0-mm-diameter and 10.0-mm-depth pTa cylinder with autologous BMSCs were implanted in subchondral bone defect sites. **e** Preparation of fibrin sealant. **f** The 10.0-mm-diameter CM with autologous chondrocytes was adhered to tantalum rods by fibrin sealant. **g** Suture of joint capsule. **h** X-ray showed the position of pTa at 16 weeks post-implantation

**Fig. 7:**
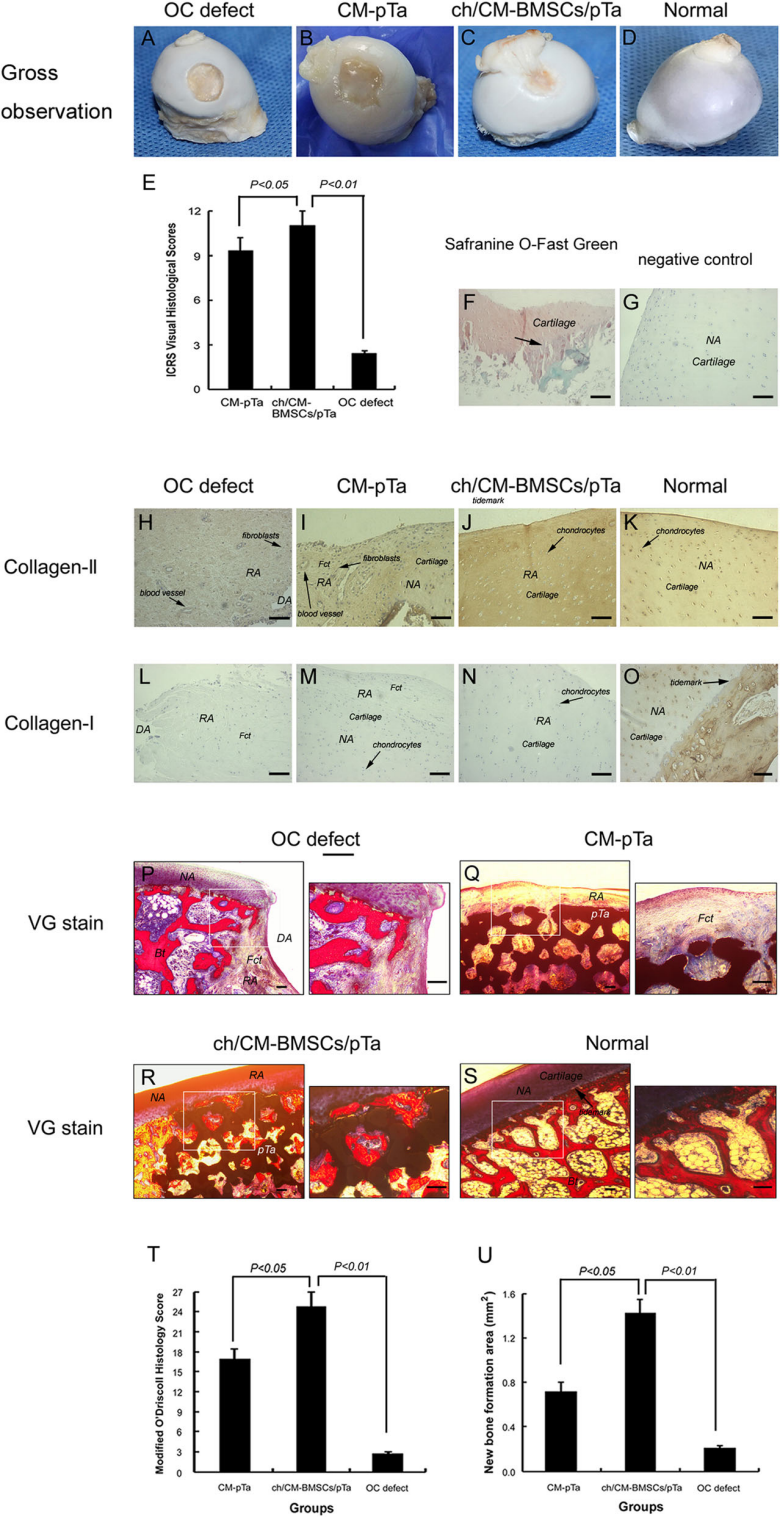
**a**–**d** Gross observation of femoral heads of goats at 16 weeks post-implantation. **e** The ICRS score for gross observation was used to assess the integration of the scaffold into the joint. **f**–**s** Histological evaluation of osteogenesis in pTa and chondrogenesis on CM at 16 weeks post-implantation. **f** Safranine O-Fast Green FCF Stain. **g** The negative control was generated by replacing the primary antibody with PBS. Immunohistochemistry of collagen II (**h**–**k**) and collagen I (**l**–**o**), bar = 100μm. **p**–**s** Hard tissue slicing and Van Gieson’s Stain at 16 weeks post-implantation, bar = 200μm. **t** Cartilage newborn was described by Modified O’Driscoll histology score. **u** New bone formation area in the pores of pTa was measured by ImageJ. Fct fibrillar connective tissue, Bt bone trabecular, pTa porous tantalum, DA defected area, RA repaired area, NA normal area

The surgical procedures to integrated repair osteochondral defects with chondrocytes/CM-BMSCs/pTa are depicted in Fig. 6a–g. X-ray images demonstrated the effect of the biphasic scaffold in Fig. 6h. In the osteochondral defect group (Fig. 7a), there was scarce repair in the defect area and no connection with the surrounding cartilage. In addition, significant degeneration of cartilage was identified. Large osteochondral defects in load-bearing areas were completely repaired at 16 weeks after operation (*n* = 8). For the CM-pTa composite (Fig. 7b), there was no infection, edema, or effusion in the surgical site 16 weeks after the operation. The articular surface in the defect area was smooth. The repaired area was tightly bound with the adjacent cartilage tissues, and no degeneration of the cartilage was observed. The normal articular cartilage was white, translucent, smooth, and glossy (Fig. 7d). Similarly, in the chondrocytes/CM-BMSCs/pTa group (Fig. 7c), the articular cartilage was slightly yellow, smooth, and elastic. The diameter of the regenerated cartilage is approximately 10.0 mm. There was no obvious boundary between the repaired area and the peripheral tissue. ICRS scores of each group for gross observation were shown in Fig. 7e.

### Histological evaluation of osteogenesis in pTa and chondrogenesis on CM

CM-pTa was implanted into osteochondral defects and formed new tissue. (Fig. 7i, m, q). The defect area was mainly filled with a large amount of vaulted fibrous tissue. We observed loose fiber structures and a layered arrangement, but there was no uniformity or transparency as observed in normal cartilage. The pores of pTa were almost entirely filled with new osteoid, and there was no obvious boundary between cartilage tissue and pTa. The newborn chondrocytes in the defect zone were immature, small, and flat with a single distribution.

The chondrocytes/CM-BMSCs/pTa group appeared similar to the normal group. Cartilage lacuna was formed in the tissue engineering scaffold. The regenerated cartilage was well integrated with the regenerated subchondral bone, and no obvious seam was observed at the interface bonding to the adjacent cartilage at both ends. Immunohistochemistry showed that the new cartilage tissue expressed collagen II (Fig. 7j) instead of collagen I (Fig. 7n), indicating that the new tissue was a cartilage tissue. Sixteen weeks after the implantation in vivo, the construct formed femoral head-like tissue with a smooth, continuous cartilage surface. Interestingly, at 16 weeks post-implantation of pTa plus autologous BMSCs, regenerated trabecular, equivalent to the mature bone, was observed in the pore of tantalum rods in defect sites. Notably, the interface between pTa and CM was uniform and smooth with firm adhesion by hard tissue slice observations (Fig. 7r).

For the osteochondral defect group, the horizontal slice demonstrated periosteum ossification (Fig. 7h, l, p). Although a small number of new bone cells were observed in bone defect sites, the fibrous connective tissue remained the main component. There were some new chondrocytes at the edges of the cartilage defect sites. But the cartilage defect area appeared to sag and was covered with fibrous tissue. Some longitudinal fibrillation was visible in the junction with repaired articular cartilage tissue. The Modified O’Driscoll Histology Score results demonstrated that chondrogenesis on CM associated with autologous chondrocytes was much better than that of implantation CM (*P* < 0.05) (Fig. 7t). Statistical results demonstrated that at 16 weeks post-implantation, pTa rods associated with autologous BMSCs accelerated the formation of new bone in the pores (*P* < 0.05) (Fig. 7u).

## Discussion

Great challenges are present with large osteochondral defects, and no “gold standard” technique currently exists for the repair of such defects. Tissue engineering has rapidly progressed from in vitro culture to in vivo implantation and has provided some possibilities for repair of large osteochondral defects and the entire articular surface. There are three major findings in this work: (1) This type of pTa which was prepared through the CVD technique on the porous SiC scaffold has no inhibitory effect on proliferation of BMSCs, chondrocytes, or MG63 cells. Meanwhile, it can promote adhesion and osteogenic differentiation of BMSCs in vitro. The implanted pTa and host bone may produce a stable connection and integration in vivo. The combination of the pTa and the BMSCs can act as a substitute for the trabecular bone in large bone defect sites. (2) Three-dimensional CM can maintain the phenotype of chondrocytes successfully and the high expression of chondrogenic genes. Cartilage defects can be successfully repaired by 3D CM combined with autologous chondrocytes; Newly formed hyaline cartilage was observed in vivo after 16 weeks of implantation. (3) An integrated repair of large osteochondral defects (10.0-mm diameter and 12.0-mm depth) in load-bearing areas in large animal was proven effective by biomimetic tissue engineering scaffolds. Meanwhile, because this kind of integrated bionic scaffold is comprised of autologous cells, injectable fibrin sealant, natural components of cartilage, and non-degradable metals with a high profile of safety, effectiveness, and simplicity, this work may provide a new tissue engineering strategy for the clinical treatment of large osteochondral lesions in the near future.

Compared with osteoblasts, BMSCs are much easier to obtain and culture in vitro. Moreover, harvesting BMSCs are less traumatic to the body. In 1987, Friedenstein et al. reported the first substitution of amplified BMSCs as “seeds” for osteoblasts to repair bone defects [28]. Many studies have reported that the bone tissue engineering complex combined with BMSCs demonstrate good osteogenic ability in animals [29, 30]. In our study, expression patterns by flow cytometry in goats are similar to BMSCs in human (Fig. 1a–h) [31]. A tri-lineage differentiation experiment was used to identify the multiple lineage differentiation capacities of the BMSCs [32]. These results suggested that our cell source used comprised identified BMSCs with multiple differentiation potential (Fig. 1i–l). Before clinical treatment, BMSCs must first be amplified in vitro to achieve sufficient quantities. The concentration of BMSCs is very important for the bone and cartilage construction in tissue engineering. Some researchers have suggested that 2.5 × 10^7^/mL is a suitable concentration for osteogenic differentiation of human mesenchymal stem cells [33]. In this study, 3 × 10^7^/mL of BMSCs were co-cultured with pTa for 3 weeks and were found to be beneficial for osteogenic differentiation. Liu et al. found that mouse embryonic stem (ES) cells differentiated on three-dimensional (3D), highly porous, tantalum-based scaffolds have significantly higher hematopoietic differentiation efficiency than those cultured under conventional two-dimensional (2D) tissue culture conditions [34]. Our Q-PCR showed that expression of bone-related genes (ALP, OSX, OCN, Col-I, OSN, and RUNX2) in 3D pTa were significantly higher than that in 2D sTa (*P* < 0.01) (Fig. 5c). So, we speculate that the three-dimensional structure and surface morphology of pTa may play an important role in promoting the differentiation of BMSCs into osteoblasts.

The proliferation of chondrocytes in the body is very limited. In this study, we found that chondrogenesis on CM associated with autologous chondrocytes was much better than that on CM alone (*P* < 0.05) (Fig. 7). Some researchers have implanted both autologous chondrocyte-seeded scaffolds and non-seeded scaffolds into two defects located in the non-load-bearing zone of the femoral condyle [35] and have found that the former is more effective, in agreement with our results. BMSCs have also been induced and differentiated into chondrocytes and used to repair cartilage defects with biomaterials [36]. However, in vivo, the progenitor cells continue to differentiate past the chondrogenic phase and proceed to hypertrophy and endochondral ossification [37]. Cell phenotype control studies have sought to determine the controlled niche required to prevent chondrocytes from fibroblast differentiation and arrest MSC differentiation at the chondrocytes stage without further progress to ossification [38, 39]. But these studies still require the use of growth factors and other exogenous substances for intervention. Therefore, the use of autologous chondrocytes from non-load-bearing areas in vitro should be a more precise and safer method for clinical application.

Besides seed cells, the choice of scaffold material is the most important factor for osteochondral repair. The engineered tissue with biphase synthetic for osteochondral repair has become one of the hot research fields. For example, Kreklau et al. placed chondrocytes into PGA/PLLA copolymer scaffolds and allowed natural coral or synthetic calcium carbonate to adhere to PGA/PLLA. This method resulted in not only a good interface between bone and cartilage but also some matrix secretion of cartilage [40]. In the study by Schaefer et al., they successfully repaired 7 mm × 5 mm × 5 mm osteochondral defects in non-load-bearing areas with the PGA/collagraft scaffold [41]. In the study of Gao et al., ICP/HA phase scaffolds made of injectable calcium phosphate and hyaluronan was used to construct the osteochondral complex [42]. The ideal artificial bone material should have a similar microstructure to that of natural cancellous bone. In addition, it should have good biocompatibility and porous interlinkages to promote the growth of blood vessels and nerves, induce bone ingrowth, and accelerate the formation of osteoid [43, 44].

Tantalum metal has good biocompatibility, ductility, and tenacity. The Zimmer company can produce pTa on a thermosetting polymer foam precursor (RVC) [45]. However, the compressive strength of RVC is poor, and it requires a thick tantalum coating to improve the mechanical properties. Some studies have found that similar RVCs on the market are cytotoxic to rabbit MSCs but not rabbit chondrocytes [46]. In this study, we used porous SiC as the scaffold and prepared tantalum coating by CVD. The compressive strength of porous SiC is better than that of RVC, so the mechanical properties are still appropriate for bone filling with a thinner tantalum coating, and consequently much lower production costs. In addition, our results demonstrated that BMSCs, chondrocytes, and MG63 cells did not suffer cytotoxicity in the presence of pTa or porous SiC scaffolds (Fig. 2). Open porosity and the pore size of porous scaffolds are the most important factors for implantation in vivo. Some studies have suggested that porous material with a pore diameter of 50–400 μm may be more beneficial to the growth of new bone [47]. In this study, the pore size of our pTa metal was approximately 200–500 μm, and open porosity was nearly 75–85%. Moreover, our pTa had sufficient mechanical strength, whereas the elastic modulus was lower than that of cortical bone and higher than that of cancellous bone. These properties of pTa make the post-implantation stress shielding and are conducive to bone remodeling [48]. Our results confirmed that our pTa can provide a safe and ideal three-dimensional spatial structure for the adhesion and proliferation of BMSCs. The osteogenic potential of pTa associated with autologous BMSCs is excellent, and the regenerated trabecular is equivalent to mature bone in the pore of the tantalum in vivo (Fig. 7c, r).

Clinical treatment of cartilage defects has been unsatisfactory for many years. Currently, many combinations of materials and different cells have been reported to repair cartilage defects. Because natural materials, such as collagen, chondroitin sulfate, and glycosaminoglycan, are components of cartilage, these have been the focus of cartilage tissue engineering. For example, Getgood et al. have used a new collagen-glycosaminoglycan-calcium phosphate biphasic scaffold for the early-stage repair of surgically created osteochondral defects in a caprine model. They have found that the biomaterial composition of the collagen-glycosaminoglycan may provide a more favorable environment for chondral repair [49]. Commercialized 3D CM are usually used after bone grafting procedures and to enhance soft tissue formation, such as reconstruction of the alveolar ridge, and cleft closure surgeries. Chondrocytes, obtained from talocrural joints of horses have been observed to form multilayers on the surface of commercialized 3D CM in vitro [50]. However, there have been few reports on commercialized 3D CM with autologous chondrocytes to repair large articular cartilage defects in large animals. In this work, chondrocytes appeared uniformly distributed on the surface and inside of the 3D CM scaffold (Fig. 3e). Chondrocytes were closely attached to the surface of the material and connected with one another. Chondrocytes proliferated quickly and completely covered the 3D CM in the extensional state. Moreover, we also found that 3D collagen membranes can promote mRNA expression of cartilage-related genes, such as Col-II, Agg, and SOX9, in goat chondrocytes compared to 2D CM (*P* < 0.01). However, 3D CM can inhibit the expression of Col-X which is related to cartilage hypertrophy (*P* < 0.01) (Fig. 5d). The fate of tissue-engineered scaffold in vivo is the most important criterion for evaluating the final osteochondral formation. We found that the 3D CM loaded with autologous chondrocytes promoted cartilage regeneration in vivo (Fig. 7c, j, n, r). This method may be applied to repair large areas of cartilage defect in clinical settings in the future.

Osteochondral defects comprise the damage of both the articular cartilage and the underlying subchondral bone. To repair an osteochondral defect, the bone, cartilage, and the bone-cartilage interface must all be considered simultaneously [6]. The articular cartilage can be divided into the superficial zone, transitional zone, deep zone, zone of calcified cartilage, and subchondral bone. The first three layers are defined as the hyaline cartilage. The tidemark is the junction of the hyaline cartilage and zone of calcified cartilage. The appearance of the tidemark is considered as cartilage maturation. The cartilage and the bone-cartilage interface which was composed of the tidemark and zone of calcified cartilage plays an important role in the structure and function of the integrated osteochondral. The material of bone-cartilage interface is a key factor to achieve an integrated repair. In the early times, surgical suture was a common method for integrated repair by tissue engineering, but it was not effective in the load-bearing areas. In this work, BMSC-loaded pTa and biomimetic 3D collagen were attached with injectable fibrin sealant clinically. We demonstrated that the interface was uniform, with smooth and firm adhesion (Fig. 3a, b). The thrombin and fibrinogen in injectable sealant combined with each other aggregated with the help of factor XIII and Ca^2+^ and formed a stable fibrin polymer. The polymer made the biomimetic 3D collagen and the BMSCs/pTa fixed firmly. Moreover, the gap between bone tissue and cartilage tissue are closed with the injectable fibrin sealant. In addition, the fibrin network provides different active substance which promotes the formation of new blood vessels and wound healing. Porcine fibrin sealant effectively fixed the combination of the bone and cartilage interface in large-area defects of load-bearing areas. Future studies should evaluate the physiological and mechanical properties of the integrated repair scaffold for longer periods of 1 or 2 years.

## Conclusion

As we know, tissue engineering scaffolds require long-term preclinical testing for decades. The safety concern is one of the most important factors during implantation and cellular therapy. In this work, an integrated repair was proved effectively by a bionic scaffold consist of autologous cells, injectable fibrin sealant, natural components of cartilage, and porous metals certified in large animal model. In vitro, this type of pTa has no inhibitory effect on proliferation of BMSCs, chondrocytes, or MG63 cells. And this kind of integrated scaffold could promote the adhesion and differentiation of autologous cells. A subsequent in vivo study found the bone and cartilage regeneration with a high profile of safety, effectiveness, and simplicity. This work demonstrated the potential for clinical transformation of the repair of large osteochondral defects in load-bearing areas of a large animal in the near future.

## Abbreviations

2D CM: Two-dimensional collagen membranes
3D CM: Three-dimensional collagen membranes
ALP: Alkaline phosphatase
BMSCs: Bone marrow mesenchymal stem cells
CM: Collagen membranes
CVD: Chemical vapor deposition
PBS: Phosphate buffer saline
PEG: Polyethylene glycol
pTa: Porous tantalum
RVC: Reticulated vitreous carbon
SEM: Scanning electron microscopy
SiC: Porous silicon carbide
sTa: Solid tantalum
